# Liposomal Delivery of MIW815 (ADU-S100) for Potentiated STING Activation

**DOI:** 10.3390/pharmaceutics15020638

**Published:** 2023-02-14

**Authors:** Nan Ji, Minjia Wang, Chalet Tan

**Affiliations:** 1 Department of Pharmaceutics and Drug Delivery, University of Mississippi, University, MS 38677, USA; 2 Department of Pharmaceutical Sciences, University of Tennessee Health Science Center, Memphis, TN 38163, USA

**Keywords:** cationic liposome, STING agonist, cancer immunotherapy

## Abstract

Stimulator of interferon genes (STING) agonists can improve the anticancer efficacy of immune checkpoint blockade by amplifying tumor immunogenicity. However, the clinical translation of cyclic dinucleotides (CDNs) as STING agonists is hindered by their poor drug-like properties. In this study, we investigated the design criteria for DOTAP/cholesterol liposomes for the systemic delivery of ADU-S100 and delineated the impact of key formulation factors on the loading efficiency, serum stability, and STING agonistic activity of ADU-S100. Our findings demonstrate that the cationic liposomal formulation of ADU-S100 can be optimized to greatly potentiate STING activation in antigen-presenting cells.

## 1. Introduction

Recent advances in cancer immunotherapy have transformed cancer medicine. Unleashing anti-tumor T cell immunity via immune checkpoint blockade can yield complete and durable responses in some patients with previously untreatable tumors, such as metastatic melanoma, providing compelling evidence that the immune system can be bolstered to combat malignancies [1,2]. The majority of cancer patients, however, are unable to achieve long-term control of cancer progression. This is largely attributable to the lack of significant T-cell infiltration in the tumor and the presence of high densities of immunosuppressive cells that inhibit anti-tumor immune responses. Overcoming and remodeling the immunosuppressive tumor microenvironment is therefore of paramount importance in order to realize the full potential of cancer immunotherapy with immune checkpoint blockade [3].

Stimulator of interferon genes (STING) has recently emerged as a promising therapeutic target to amplify tumor immunogenicity and enhance the rates at which patients respond to immune checkpoint inhibitors [4,5]. STING is a critical adaptor protein that mediates innate immune sensing of cancer [6]. In response to cytosolic DNA shed by tumor cells, 2′3′-cyclic guanosine monophosphate-adenosine monophosphate (cGAMP), a secondary messenger in eukaryotic cells and an endogenous ligand for STING, is produced to bind STING and trigger the activation of the STING signaling pathway that leads to the upregulation of type I interferon, a key prerequisite for the maturation of dendritic cells in the tumor microenvironment and the ensuing anti-tumor immune responses. STING can also be activated by bacteria-derived cyclic dinucleotides (CDNs) such as c-di-AMP and c-di-GMP [7]. However, the chemical features of natural CDNs restrict their use to intratumoral administration: CDNs are prone to instant enzymatic hydrolysis by the phosphodiesterase ubiquitous in tissues and blood; their anionic and polar structures diminish membrane permeability and cellular uptake.

MIW815 (ADU-S100, also known as ML-RR-S2-CDA) is a bisphosphothioate analog of cyclic di-AMP. The substitution of the non-bridging oxygen atoms at the internucleotide phosphate bridge with sulfur atoms makes ADU-S100 less susceptible to enzymatic hydrolysis by ecto-nucleotide pyrophosphatase/phosphodiesterase (ENPP1) [8]. The phosphate bridge configuration of ADU-S100 contains both 2′-5′ and 3′-5′ linkages, resulting in enhanced binding affinity to STING and more potent proinflammatory responses compared with cGAMP. In a landmark study by Corrales et al., intratumoral injection of ADU-S100 was found to cause the regression of pre-existing tumors in mice and generate systemic immune responses with sustained immunological memory [9]. In another interesting study, ADU-S100 was formulated with granulocyte-macrophage colony-stimulating factor-producing cellular cancer vaccine (STINGVAX), which was injected subcutaneously into the contralateral limb of tumor-bearing mice, leading to potent anti-tumor immunity against multiple aggressive murine tumors [10]. When combined with PD-1 blockade, STINGVAX induced the regression of poorly immunogenic tumors that were resistant to PD-1 blockade alone. With very promising preclinical results, ADU-S100 was advanced to phase I/II clinical trials (NCT02675439, NCT03172936, and NCT03937141). ADU-S100 was found to be readily absorbed from the injection site and rapidly eliminated from the bloodstream with a terminal half-life of approximately 24 min. Although well tolerated in patients, the clinical responses to intratumoral ADU-S100 treatment were less robust than expected [11]. A multitude of factors may have contributed to the modest therapeutic outcomes, one of which may be the suboptimal dosing route. Intratumoral treatment provides a direct approach to evaluate safety and anti-tumor effect. However, this administration route results in inconsistent drug distribution and can only be adopted for patients with accessible cancer types. Therefore, developing safe and effective strategies for the systemic delivery of CDNs is of high clinical significance, in particular for the treatment of metastatic cancer.

The therapeutic promise of CDNs seen in murine models has motivated intense efforts to explore strategies that enable intravenous delivery of cGAMP, ADU-S100, and other CDNs. A variety of nanocarrier systems ranging from polymeric to metal-based nanoparticles have been devised to improve the stability and cytosolic delivery of CDNs [12,13,14,15,16,17]. As one of the most clinically relevant nanoformulations, the use of liposomal nanocarriers has been pursued by several groups. A well-studied cationic lipid, 1,2-dioleoyl-3-trimethylammonium-propane (DOTAP), was used to generate positively charged liposomes that entrapped anionic CDNs via charge–charge interaction. In a study pioneered by Koshy et al., a phosphorothioated cGAMP analog was encapsulated in PEGylated DOTAP-based liposomes, which allowed the systemic delivery of cGAMP to a metastatic B16-F10 melanoma in the lung, resulting in reduced tumor growth, while a free drug at the same dose produced no effect [18]. Using both orthotopic and genetically engineered mouse models of basal-like triple-negative breast cancer, Cheng et al. reported impressive innate and adaptive immune responses to pre-existing tumors following intravenous administration of a DOTAP-containing liposomal formulation of cGAMP, whereas the effect of the free drug was again negligible [19]. More recently, ADU-S100 was encapsulated in DOTAP-based cationic liposomes that were functionalized with Clec9a peptide to target CD103^+^ dendritic cells [20]. Intravenous treatment with this targeted liposomal formulation yielded potent anti-tumor efficacy accompanied by robust immune responses both as a monotherapy and in combination with anti-PD-L1 antibody therapy in mice bearing B16-F10 and MC38 tumors.

Despite the promising anticancer efficacy demonstrated by liposomal CDNs in the above studies, from the standpoint of formulation development, details concerning the key parameters for the preparation of liposomal CDNs were lacking. In this work, we aimed to investigate the design criteria for the DOTAP-based liposomal delivery of ADU-S100. In particular, we focused on the optimization of the charge ratio between DOTAP and ADU-S100 based on its effect on the loading efficiency, serum stability, and STING agonistic activity of ADU-S100. Furthermore, PEGylation of liposomal ADU-S100 was shown to be another critical factor that not only influenced the stability but also the potency of ADU-S100. Such insights linking the physicochemical properties of formulations to STING agonism in target cells can inform the design criteria for cationic liposomes with broad applicability to other CDNs.

## 2. Materials and Methods

### 2.1. Chemicals

ADU-S100 was purchased from Chemietek (Indianapolis, IN, USA) and cyclic AMP (cAMP) was purchased from Cayman Chemical (Ann Arbor, MI, USA). All of the following lipids were purchased from Avanti Polar Lipids (Alabaster, AL, USA): 1,2-dioleoyl-3-trimethylammonium-propane (chloride salt) (18:1 TAP (DOTAP)), hydrogenated soy L-α-phosphatidylcholine (HSPC), cholesterol, and 1,2-distearoyl-sn-glycero-3-phosphoethanolamine-*N*-[methoxyl (polyethylene glycol)-2000] (ammonium salt) (DSPE-PEG_2000_). All other chemicals were of analytical grade.

### 2.2. Cell Culture

THP-1 Dual cells (InvivoGen, San Diego, CA, USA) were cultured in RPMI 1640 medium (ATCC, Manassas, VA, USA) supplemented with glutamine, penicillin (100 U/mL), streptomycin (100 µg/mL), normocin (100 µg/mL), and 10% heat-inactivated fetal bovine serum. To maintain the selection pressure, blasticidin (10 µg/mL) and zeocin (100 µg/mL) were added to the growth medium every other passage.

Bone marrow cells were isolated from femurs and tibias of 8–12-week-old C57BL/6 mice and seeded in a 10-cm petri dish at a density of 2 × 10^6^ viable cells in 10 mL RPMI 1640 medium supplemented with glutamine, penicillin (100 U/mL), streptomycin (100 µg/mL), 2-mercaptoethanol (55 µM), 10% heat-inactivated fetal bovine serum, and 20 ng/mL of murine granulocyte-macrophage colony-stimulating factor (GM-CSF, Peprotech). An additional 10 mL of fresh warm medium with GM-CSF (40 ng/mL) was added on day 3 and a half volume of fresh medium was exchanged for fresh warm medium with GM-CSF (40 ng/mL) on day 6. On day 8, non-adherent and loosely adherent cells in the culture supernatant were harvested. The percentage of CD11c^+^ bone marrow-derived dendritic cells (BMDCs) was approximately 70%, as determined by flow cytometry. More concentrated CD11c^high^ BMDCs were positively selected by mouse CD11c^+^ microbeads ultrapure (Miltenyi Biotec, Bergisch Gladbach, Germany).

### 2.3. Preparation and Characterization of Liposomal ADU-S100

To prepare ADU-S100-loaded liposomes, a total of 3 µmol of a lipid mixture of DOTAP, HSPC, cholesterol, and DSPE-PEG_2000_ was dissolved in ethanol at different molar ratios, as shown in Table 1. This was then dried under a rotary evaporator to yield a thin lipid film. The resulting lipid film was further dried in a vacuum desiccation chamber overnight. The dry film was hydrated above 55 °C in a solution of ADU-S100 (125 µg/mL, 170 µM) in HEPES buffer (10 mM, pH 7.4) by vigorous mixing. The hydrated solution was then placed in a sonication bath for 15 min above 55 °C and filtered using a 100-KD MWCO centrifugal filter (Amicon) to remove the free drug. Liposomes exteriorly complexed with ADU-S100 (denoted as MIX lipo ADU-S100) were prepared by directly mixing ADU-S100 with pre-formed blank liposomes as a control formulation. The concentration of ADU-S100 in the resultant liposomal formulations was determined using high-performance liquid chromatography (HPLC) as described below. The ADU-S100 loading efficiency was calculated as the percentage of the incorporated vs. the input drug.

The hydrodynamic diameter and the zeta potential of the liposomes were measured using a Malvern Zetasizer Nano ZS (Southborough, MA, USA). The liposomes were diluted 20-fold in NaCl solution (10 mM) and analyzed at 25 °C using a quartz cuvette with a minimum of three measurements for each sample.

### 2.4. HPLC Analysis of ADU-S100

A sensitive and robust HPLC analytical method was developed for the in vitro quantification of both the liposome-encapsulated ADU-S100 and the free ADU-S100. Drug-loaded liposome samples were lysed with Triton-X100 (0.1%, *v*/*v*) to release the encapsulated content. For the serum samples, five times (by volume) of acetonitrile was used to disrupt the liposome and precipitate the serum proteins, with cyclic adenosine monophosphate (cAMP) spiked as an internal standard. Following centrifugation, the supernatant was dried in a SpeedVac vacuum concentrator and was then reconstituted in the mobile phase for HPLC analysis. An Agilent 1260 Infinity II HPLC system (Palo Alto, CA, USA) equipped with an autosampler, a PDA detector, and an OpenLAB CDS computer system was used. An Agilent Poroshell 120 HILIC-z column (2.7 μm, 3 × 150 mm) with a guard column (3.5 μm, 3 × 3 mm) was used. An isocratic elution method was applied with a mobile phase of 91% A (90% acetonitrile and 10% ammonium acetate buffer (pH = 9, 100 mM)) and 9% B (ammonium acetate buffer, pH = 9, 10 mM) at a flow rate of 0.5 mL/min. The UV absorbance wavelengths for the ADU-S100 and cAMP were 258 nm and 256 nm, respectively. A representative HPLC chromatogram can be found in Figure S1.

### 2.5. Serum Stability Study

At 37 °C, liposomal ADU-S100 or the free drug was incubated with fetal bovine serum at a 1:10 ratio (*v*/*v*) with a final ADU-100 concentration of 40 µM. The serum samples were collected at pre-determined time points and quantified for ADU-S100 using the HPLC method described above.

### 2.6. THP1-Dual Reporter Assay

THP-1 Dual cells were used to determine the interferon-regulatory factor 3 (IRF3) pathway by monitoring the activity of Lucia luciferase, and the NF-κB pathway by assessing the activity of secreted embryonic alkaline phosphatase (SEAP). THP-1 Dual cells were seeded in 96-well flat-bottom culture plates at 75,000 cells per well in 200 μL of complete RPMI 1640 growth media and treated with various liposomal ADU-S100 formulations and free ADU-S100. PBS was used as a negative control. After 48 h of incubation, the culture medium was collected and analyzed for its luciferase and SEAP levels. Briefly, 20 µL of cell culture supernatant was transferred into a white opaque 96-well plate, 50 µL of QUANTI-Luc™ assay solution was added to each well, and the luminescence was recorded using a Synergy H1 plate reader (BioTek, Winooski, VT, USA). In addition, 50 µL of cell culture supernatant was added to 150 µL of QUANTI-Blue SEAP detection medium and incubated for 2 h at 37 °C, and the absorbance at 620 nm was measured using a plate reader. The dose–response curves of the reporter assays from three independent experiments were plotted using GraphPad Prism (San Diego, CA, USA). The EC_50_ was calculated using GraphPad Prism and presented as average ± SD.

### 2.7. Maturation of Dendritic Cells by Flow Cytometry Analysis (FACS)

BMDCs were plated at 2 × 10^6^ cells per well in a 24-well non-treated tissue culture plate and stimulated with liposomal ADU-S100 (0.5 µg/mL) or free ADU-S100 (5 µg/mL) for 6 h. Cells were collected and washed with FACS buffer via centrifugation for 3 min at 500 g. Cells were treated via Fc block and incubated at 4 °C for 30 min. They were then washed with FACS buffer (2% FBS, 2.5 mM EDTA in 1X HBSS) and stained with CD11c-APC (N417), MHCII-PerCP-Cy5.5 (M5/114.15.2), CD40-PE (3/23), CD80-PE (16-10A1), and CD86-PE (GL-1) at 4 °C for 30 min. All antibodies were purchased from BioLegend unless otherwise stated. The cells were washed and a FACS analysis was performed using Guava EasyCyte (Luminex, Austin, TX, USA). Flow cytometer configurations and compensation settings were optimized using unlabeled and single color-stained samples. All flow cytometry raw data were analyzed using FlowJo software (version 10). The gating strategies can be found in Figure S2.

### 2.8. Quantification of IFNβ and TNFα Using ELISA

BMDCs were plated at a density of 0.5 × 10^6^ cells/mL in a 96-well plate and treated with various liposomal formulations or free ADU-S100 for 24 h. The cell culture supernatant was collected and the concentrations of IFNβ and TNFα were determined using ELISA (R&D, Minneapolis, MN, USA) according to the manufacturer’s protocols.

## 3. Results

### 3.1. Synthesis and Characterization of Liposomal ADU-S100

**Figure 1:**
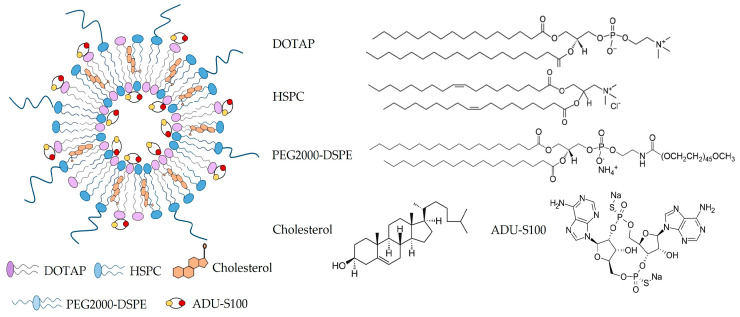
Schematic representation of ADU-S100-loaded liposome and the chemical structures of the individual components. DOTAP, HSPC, and PEG_2000_-DSPE are assembled into the lipid bilayer, and cholesterol is inserted within the lipid bilayer. ADU-S100 can be associated with both the interior and exterior of the liposome bilayer by complexing with the cationic amino headgroup in DOTAP.

**Figure 2:**
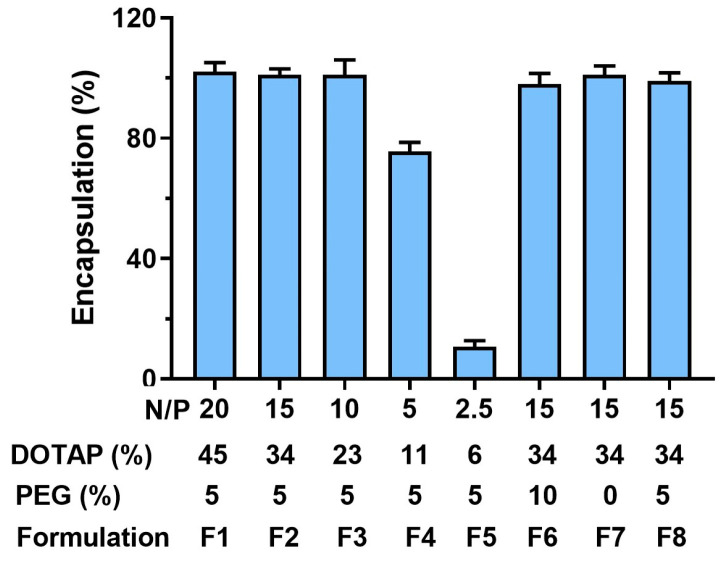
Loading efficiency of liposomal ADU-S100 formulations with varying levels of DOTAP and PEGylation. Data are shown as the mean ± SD (*n* ≥ 3) and are representative of three independent formulation preparations.

The scheme of ADU-S100-loaded liposome is shown in Figure 1. The total lipid concentration in all the liposomal formulations was kept at 15 mM with a total of 3 µmol of lipids in 200 µL of HEPES, which contained varying percentages of DOTAP (6–45 mol%), DSPE-PEG_2000_ (0–10 mol%), and HSPC (0–39 mol%), as is shown in Table 1. In addition, 50 mol% cholesterol was included in all the liposomes. In the drug-loaded liposomes, 125 µg/mL of ADU-S100 (170 µM in HEPES) was used to hydrate the lipid films. The N/P ratio, which is the molar ratio between the cationic amine (NH_3_^+^) groups in the DOTAP and the anionic phosphates (PO_4_^3−^) groups of the ADU-S100, was calculated to correlate with the loading efficiency, the particle size, and the zeta potential of the liposomal ADU-S100 [21]. We found that liposomes with an N/P ratio of 10:1 (23 mol% DOTAP) or higher could achieve close to 100% loading efficiency, whereas, at an N/P ratio of 2.5:1 (6 mol% DOTAP), the loading efficiency was only 10% (Figure 2). These results suggest that the electrostatic interaction between ADU-S100 and DOTAP drives the drug’s incorporation into DOTAP/cholesterol liposomes, and an excessive positive charge beyond the charge-neutralization point is required for the efficient loading of ADU-S100. We hypothesized that negatively charged ADU-S100 molecules could be complexed with the positively charged DOTAP molecules on both the exterior and interior of the lipid bilayer (Figure 1). To test this hypothesis, MIX lipo ADU-S100 was prepared as a control formulation by directly mixing ADU-S100 with the pre-formed liposomes (34 mol% DOTAP, 5 mol% PEG) in which the ADU-S100 molecules were only bound to the DOTAP present on the exterior of the liposomal membrane. Interestingly, MIX lipo ADU-S100 (F8) had a near 100% incorporation efficiency following centrifuge filtration to remove any free molecules, showing that ADU-S100 can be stably complexed onto the DOTAP/cholesterol liposomal surface.

**Figure 3:**
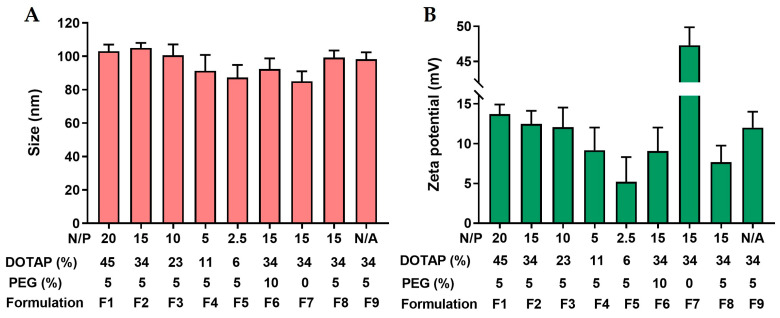
Physical characterization of liposomal ADU-S100 formulations with varying levels of DOTAP and PEGylation. The average hydrodynamic diameter (**A**) and zeta potential (**B**) of the liposomal ADU-S100 were determined using Nano Zetasizer. Data are shown as the mean ± SD (*n* ≥ 3) and are representative of three independent formulation preparations.

Upon rehydration of the thin lipid film, the dispersion of the lipid components and the ADU-S100 in the aqueous solution was expected to form large multilaminar lipid vesicles with a broad size range. Smaller unilaminar liposomes with a narrower size distribution were obtained following bath sonication. As is shown in Figure 3A, the resulting liposomal formulations presented a similar dynamic size, ranging from 85 nm to 105 nm, with a PDI below 0.2, indicating a homogenous population and a narrow size distribution of DOTAP/cholesterol liposomes. The impact of the DOTAP and PEGylation on the zeta potential of the liposomes is summarized in Figure 3B. When PEGylation was set at 5 mol% (F1–F5), the zeta potential of the liposomes was dependent on the DOTAP content; the zeta potential gradually increased and reached a plateau of 13–14 mV when DOTAP was above 23 mol%. When DOTAP was kept at 34 mol% (F2, F6, F7), the zeta potential of the liposomes was inversely correlated with the PEG content, with non-PEGylated liposomes (F7) being highest at 47 mV and 10 mol% PEGylated liposomes (F6) being lowest at 10 mV. Compared with F2, MIX lipo ADU-S100 (F8) had somewhat reduced zeta potential, likely due to the additional charge neutralization resulting from the ADU-S100 being complexed exclusively onto the exterior of the liposome membrane. The tabulated size distribution and zeta potential information for all the ADU-S100-loaded liposomal formulations can be found in Table S1.

### 3.2. Formulation Factors Influencing the Serum Stability of Liposomal ADU-S100

**Figure 4:**
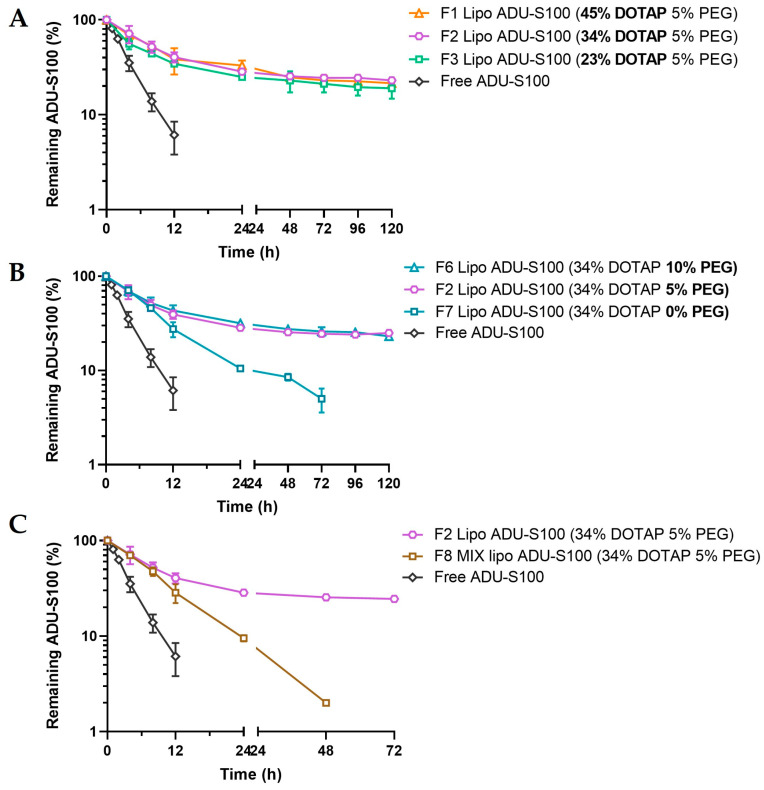
Formulation factors influencing the serum stability of liposomal ADU-S100. Effects of DOTAP (**A**), PEGylation (**B**), and the drug loading method (**C**). Liposomal ADU-S100 formulations were incubated in fetal bovine serum at 37 °C, and the concentration of AUD-S100 was quantified at the indicated time points using HPLC. Data are shown as the mean ± SD (*n* ≥ 3).

Liposomes are known to be susceptible to lipoprotein-induced leakage in serum, and cationic liposomes in particular tend to aggregate with serum proteins that are negatively charged [22,23]. It is therefore important to evaluate and improve the serum stability of the liposome formulations. ADU-S100, a phosphorothioated c-di-AMP with enhanced resistance against hydrolysis, is still prone to enzymatic degradation in serum. As is shown in Figure 4A, free ADU-S100 readily underwent a monoexponential decline in serum with a half-life of 2.8 h. Over 90% of the drug was degraded within 12 h, and it became undetectable by 24 h. When PEGylation was kept at 5 mol%, liposomal formulations with 23 mol% or higher DOTAP significantly improved the serum stability of the ADU-S100, with over 30% of the drug remaining in the serum at 72 h. However, there was no additional protective effect when further increasing the DOTAP content. To evaluate the effect of PEGylation on the serum stability of liposomal ADU-S100, formulations with identical DOTAP (34 mol%) and varying PEG (0–10 mol%) were studied. We found that ADU-S100 loaded in non-PEGylated liposomes was significantly less stable in serum than those loaded in the PEGylated liposomes, and liposomal ADU-S100 with 5 mol% or 10 mol% PEGylation protected the ADU-S100 from serum degradation to a similar extent (Figure 4B).

PEGylated liposomal ADU-S100 exhibited biexponential degradation kinetics in serum, with a rapid decline of about half of the drug within the first 8 h and a much slower decline over the next several days. We speculated that the initial rapid decline was due to the fast disassociation of ADU-S100 from the liposome exterior, which was susceptible to enzymatic degradation in serum, whereas the encapsulated ADU-S100 within the liposome interior was protected from the serum, resulting in persistent ADU-S100 levels. Interestingly, MIX lipo ADU-S100 (F8) showed fast degradation kinetics that closely resembled the rapid decline phase observed in liposomal ADU-S100 (F2) during the initial 8 h, supporting the notion that the initial rapid degradation of liposomal ADU-S100 is likely due to the loss of surface-bound ADU-S100 (Figure 4C). We reasoned that since all the drug molecules in the MIX lipo ADU-S100 (F8) were bound to the liposome surface, the degradation of ADU-S100 was quick and followed first-ordered kinetics (i.e., monoexponential). Together, these results indicate that encapsulation of ADU-S100 within PEGylated DOTAP/cholesterol liposomes can markedly improve the serum stability of ADU-S100.

### 3.3. Formulation Factors Influencing STING Activation by Liposomal ADU-S100

**Figure 5:**
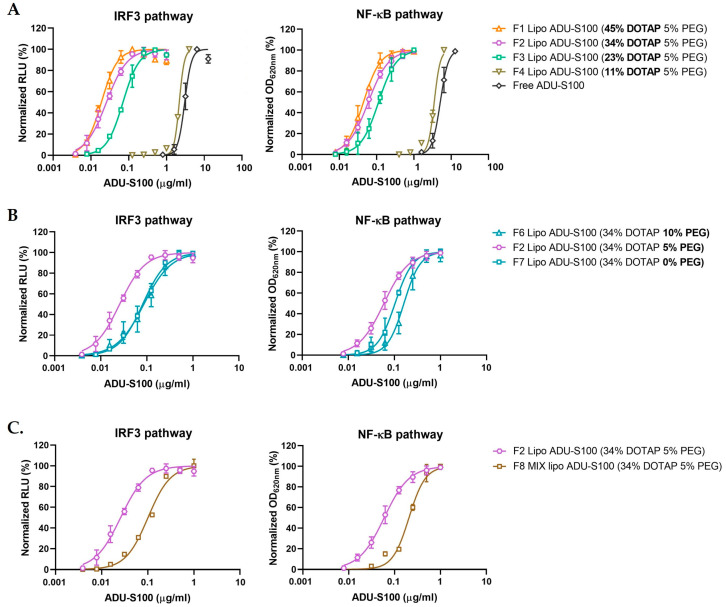
Formulation factors influencing STING activation by liposomal ADU-S100. Effects of DOTAP (**A**), PEGylation (**B**), and the drug loading method (**C**). THP-1 Dual cells were stimulated with free ADU-S100 or liposomal ADU-S100 formulations for 48 h. The cell culture medium was collected to determine the activation of the IRF3 and NF-κB pathways by measuring the activities of Lucia luciferase and SEAP, respectively. The connecting lines are variable-slope dose–response curve fits. RLU: relative light units; OD: optical density. Data are shown as the mean ± SD (*n* ≥ 3) and are representative of three independent experiments.

To evaluate the intracellular STING activation by liposomal ADU-S100, we employed THP-1 Dual cells that allow for the simultaneous monitoring of the IRF3 and NF-κB pathways downstream of the STING signaling. THP-1 Dual cells were incubated with a series of liposomal ADU-S100 formulations with varying DOTAP and PEGylation. As indicated by the dose–response curves in Figure 5A and the EC_50_ values in Table 2, free ADU-S100 induced the IRF3-mediated luciferase and NF-κB-mediated SEAP expression at half maximum effective concentration (EC_50_) values of 3.03 µg/mL and 4.85 µg/mL, respectively. Strikingly, the liposomal ADU-S100 with 5 mol% PEGylation potentiated STING activation in THP-1 cells by two orders of magnitude when 34 mol% or 45 mol% of DOTAP was used. By contrast, liposomal ADU-S100 containing 23 mol% DOTAP was noticeably less potent, and the liposomes with 11 mol% DOTAP failed to significantly shift the dose–response curves compared with those of free ADU-S100. These results indicate that the DOTAP level in liposomes determines the potency of ADU-S100. Next, we sought to evaluate whether there was an optimal PEGylation level for liposomal ADU-S100 to maximize STING activation. Since there was no significant difference between the liposomes with 34 mol% and 45 mol% DOTAP in terms of the IRF3- and NF-κB-mediated reporter activities, we focused on liposomal ADU-S100 containing 34 mol% DOTAP with varying PEG levels (0–10 mol%) to study the effect of PEGylation on STING activation. We observed that 5 mol% PEGylated liposomal ADU-S100 produced the most potent responses. Non-PEGylated liposomal ADU-S100 produced responses similar to those of 10 mol% PEGylated liposomal ADU-S100 (Figure 5B). MIX lipo ADU-S100 (F8) resulted in much weaker STING activation than liposomal ADU-S100 (F2), indicating that ADU-S100 encapsulation was responsible for the efficient STING activation (Figure 5C). It is also worth noting that liposomal ADU-S100 containing 45 mol% DOTAP caused notable cytotoxicity at a lipid concentration of 250 μM, whereas the formulations with 34 mol% or less DOTAP showed minimal cytotoxicity (Figure S3).

**Figure 6:**
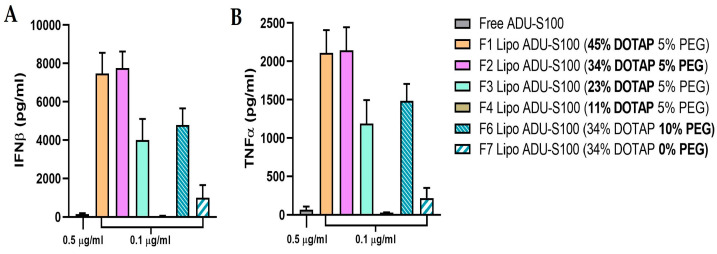
Liposomal ADU-S100 enhances TNFα and IFNβ production. BMDCs were treated with free ADU-S100 (0.5 µg/mL) or liposomal ADU-S100 (0.1 µg/mL) for 24 h, and the IFNβ (**A**) and TNFα (**B**) levels in the cell culture medium were determined using ELISA. Data are shown as the mean ± SD (*n* ≥ 3) and are representative of three independent experiments.

To further assess the impact of formulation factors on the biological activity of liposomal ADU-S100, the levels of IFNβ and TNFα, two well-known downstream targets of STING activation, were quantified in BMDCs (Figure 6 and Figure S4). Consistent with the trend observed with the reporter assays in THP-1 Dual cells, 5 mol% PEGylated liposomes with 34 mol% (F2) or 45 mol% DOTAP (F1) carrying ADU-S100 (0.1 µg/mL) increased the production IFNβ 50-fold and the production of TNFα 33-fold compared with the free drug (0.5 µg/mL), whereas lowering DOTAP to 23 mol% (F3) halved the levels of both cytokines. The PEGylation of liposomal ADU-S100 formulations affected the cytokine production in BMDCs. Liposomal ADU-S100 with 10 mol% PEG (F6) induced significantly lower cytokine production than that with 5 mol% PEG (F2), likely reflecting the steric hindrance of the PEG coating interfering with the cellular uptake of DOTAP/cholesterol liposomes. Noticeably, non-PEGylated liposomal ADU-S100 (F7) caused much lower production of TNFα and IFNβ, a finding consistent with the poor stability of the non-PEGylated liposomes observed in the serum-containing culture medium (Figure 4B).

### 3.4. Liposomal Delivery of ADU-S100 Augments the Maturation of Bone Marrow-Derived Dendritic Cells (BMDCs)

**Figure 7:**
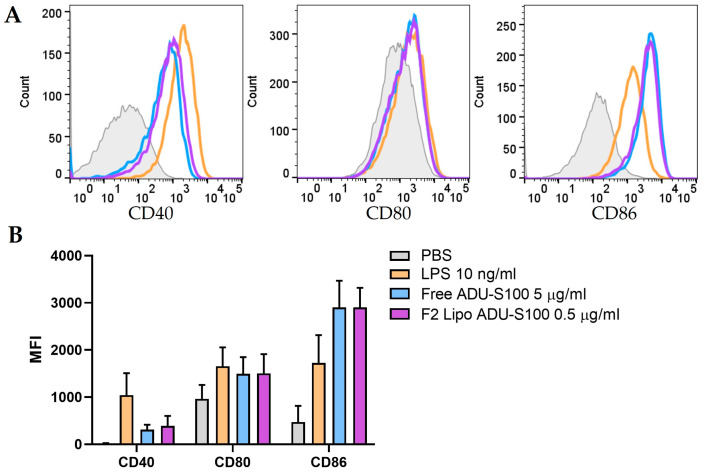
Liposomal ADU-S100 promotes the maturation of dendritic cells. (**A**) Representative flow cytometry histograms of CD40, CD80, and CD86 expression after the stimulation of BMDCs with free ADU-S100 (5 µg/mL) or liposomal ADU-S100 (0.5 µg/mL) for 6 h. LPS (10 ng/mL) was used as a positive control. (**B**) Mean fluorescence intensity (MFI) for CD40, CD80, and CD86 are summarized with bar graphs. Data are shown as the mean ± SD (*n* ≥ 3) and are representative of three independent experiments.

The activation of the STING pathway is known to promote the maturation of dendritic cells, which is critical for initiating an anti-tumor immune response [24,25]. To further evaluate the biological functions of liposomal ADU-S100, we examined the surface markers of dendritic cell activation and maturation in BMDCs using flow cytometry analysis. We focused on DOTAP/cholesterol liposomes with 34 mol% DOTAP (N/P ratio: 15) and 5 mol% PEGylation, as this formulation of ADU-S100 exhibited the most promising profiles in terms of serum stability (Figure 4) and STING agonism (Figure 5 and Figure 6). A potent TLR4 agonist, LPS (10 ng/mL), was used as a positive control. As is shown in Figure 7, the expression of CD40, CD80, and CD86 on the surface of BMDCs was notably upregulated following 6 h of incubation with free ADU-S100 at 5 μg/mL, but not at 0.5 µg/mL (Figure S5). However, similar levels of upregulation were observed with liposomal ADU-S100 at 0.5 μg/mL, strongly suggesting that liposomal ADU-S100 can potentiate the activation and maturation of antigen-presenting cells.

## 4. Discussion

Liposomes are historically the most successful drug delivery systems that have been employed to formulate a wide variety of therapeutic agents owing to their high degree of biocompatibility and versatility. Currently, there are 18 FDA-approved liposomal drugs in clinical use, the majority of which are for intravenous administration [26]. Doxil, the very first liposomal formulation approved by the FDA in 1995, is liposomal doxorubicin that contains HSPC, cholesterol, and DSPE-PEG_2000_ at a 56:38:5 molar ratio. This formulation is the result of nearly two decades of optimization to ensure stable and efficient drug encapsulation, fewer unfavorable interactions with serum proteins, a longer circulation time, and fewer dose-limiting toxicities [27]. One of the most critical structural components in the mammalian cell plasma membrane, cholesterol accounts for about 30–50 mol% of the entire lipid compounds in the cell membrane. The incorporation of cholesterol into the liposomal membrane not only improves the phospholipid packing and the membrane strength, but also reduces serum protein binding, minimizing the premature leakage of enclosed drug molecules in the bloodstream [28]. PEGylation of the liposomes by inserting PEG-lipids into the lipid bilayer shields the liposomal surface from aggregation, protein adsorption, opsonization, and phagocytosis. PEGylated liposomes, sometimes referred to as sterically stabilized liposomes or “stealth” liposomes, usually contain a PEG moiety of 2 KD at a surface density of 5–10 mol% to achieve a prolonged circulation time [29].

Delivering nucleic acids to intracellular sites of action is challenging because of their anionic, hydrophilic, and unstable structures. The first cationic lipid for transfection, *N*-[1-(2,3-dioleyloxy)propyl]-*N*,*N*,*N*-trimethylammonium chloride (DOTMA), was shown to transfect plasmid DNA into cells in 1987 [30]. The use of DOTAP, an ester analog of DOTMA, as a transfection reagent was introduced in 1988 [31]. The chemical modification of replacing the ether bond with an ester bond adds to the biodegradability of DOTAP and significantly reduces its cytotoxicity compared with DOTMA and other non-degradable lipids. DOTAP-based liposomes are commonly studied for the intracellular delivery of nucleic acids, such as plasmid DNA, mRNA, and oligonucleotides [32,33,34,35]. The use of cholesterol as a helper lipid renders DOTAP/cholesterol liposomes resistant to the destabilizing effects caused by the serum proteins. At a molar ratio of 1:1, DOTAP/cholesterol liposomes have demonstrated highly efficient DNA delivery and transgene expression in the lung following intravenous injection in mice [36,37]. These promising preclinical results led to a phase I clinical trial of DOTAP/cholesterol liposomes encapsulating a *TUSC2* expression plasmid in patients with recurrent and/or metastatic lung cancer. Although a small-scale trial with only 8 patients enrolled, this first-in-human study demonstrated that DOTAP/cholesterol liposomes can be safely administered to patients intravenously and resulted in the uptake of the *TUSC2* gene by human primary and metastatic tumors as well as anti-tumor effects [38].

To achieve the systemic delivery of ADU-S100 using DOTAP/cholesterol liposomes, there are several important considerations: (i) ADU-S100 should be efficiently and stably incorporated into the liposomes; (ii) liposomal ADU-S100 should protect the payload from spontaneous degradation in blood; (iii) liposomal ADU-S100 should retain potent STING agonistic activity. Being a dinucleotide analog, ADU-S100 is a small molecule that can form only two electrostatic bonds with DOTAP. Given the essential role of electrostatic interaction between DOTAP and ADU-S100, one key parameter to optimize for the liposomal formulation is the charge ratio between these two molecules, i.e., the N/P ratio. We observed that a minimum N/P ratio of 10 was required for ADU-S100 to be complexed with DOTAP/cholesterol liposomes at 100% loading efficiency. This N/P ratio threshold is notably higher than those for oligonucleotides (such as siRNA) or polynucleotides (such as plasmid DNA) delivered by DOTAP-based liposomes or other cationic nanocarriers [39,40]. It underscores a major distinction that sets the formulation of CDNs apart from those of the larger-sized nucleic acids. Instead of multivalent charge–charge interactions that mediate the stable complexation of oligo- or polynucleotide chains onto the cationic vehicles, CDNs can only form divalent bonds, and a large excess of cationic charges appears to be necessary to make up for the deficient electrostatic forces. By dissolving the drug in the aqueous buffer used to hydrate the thin lipid film containing the DOTAP and helper lipids (cholesterol, HSPC, DSPE-PEG_2000_), ADU-S100 can be encapsulated in PEGylated DOTAP/cholesterol liposomes (N/P ratio ≥ 10) that remain partially stable in serum for days. The optimization of the N/P ratio also needs to be balanced with safety considerations because DOTAP, as a cationic lipid, can non-specifically bind to anionic plasma membranes and cause dose-dependent cytotoxicity [41,42]. Given the nearly identical encapsulation and bioactivity profiles observed for liposomal ADU-S100 with 45 mol% (N/P ratio = 20) and 34 mol% DOTAP (N/P ratio = 15), we chose to focus on the later formulation to minimize potential cytotoxicity.

Apart from the N/P ratio requirement, the PEGylation of DOTAP/cholesterol liposomes was shown to be another critical factor for ensuring the stability of liposomal ADU-S100 in serum. Cationic liposomes and nanoparticles are prone to bind with anionic serum proteins as well as with cell membranes known to be rich in negatively charged glycoproteins, leading to quick aggregation and their removal from circulation [43]. In this study, a PEG coating was shown to shield the cationic charge and reduce the surface potential of the DOTAP/cholesterol liposomes, greatly improving the colloidal stability in serum. When evaluating the serum stability of liposomal ADU-100 with 5 mol% or 10 mol% PEGylation, we observed consistently that around 40% of the payload remained intact in serum for at least 3–5 days, although about half of the drug quickly underwent degradation during the first several hours. This may be explained by the quick hydrolysis of ADU-S100 at the exterior surface of liposomes in the presence of serum nucleases. On the other hand, ADU-S100 encapsulated within the PEGylated liposomes can be protected from enzymatic degradation. To develop an optimized DOTAP-based liposomal ADU-S100 formulation, it is therefore critical to validate the choices of N/P ratio and PEGylation level as these are the two basic design criteria.

In addition to the encapsulation and stability considerations for liposomal ADU-S100, another important aspect of the formulation design is to delineate how the formulation parameters affect the STING activation potency. By employing THP-1 Dual cells expressing two reporters downstream of the STING signaling, we were able to quantify the STING agonistic activity of liposomal ADU-S100 with varying N/P ratios and PEGylation levels. We found that the formulation of ADU-S100 in DOTAP/cholesterol liposomes with 34 mol% DOTAP (N/P ratio = 15) and 5 mol% PEG significantly potentiated the STING activity by two orders of magnitude, whereas further increasing the DOTAP content barely shifted the dose–response curves, suggesting a plateau of maximum potentiation by DOTAP/cholesterol liposomes. On the other hand, we observed a clear reduction in the drug potency when the PEGylation of DOTAP/cholesterol liposomes was raised from 5 mol% to 10 mol%. This is likely due to the decreased cellular uptake of the liposomes, as PEGylation can impede the binding of cationic liposomes with the cytoplasm membrane [18]. To optimize the PEGylation level for liposomal ADU-S100, it is necessary to balance the competing needs for long-circulating stability and internalization into the target cells, which will require in-depth pharmacokinetic and pharmacodynamic studies in vivo.

## 5. Conclusions

Repurposing DOTAP/cholesterol liposomes for systemic delivery of ADU-S100 requires the optimization of several key formulation factors. Liposomal ADU-S100 holds great translational potential to augment STING activation in antigen-presenting cells.

## Figures and Tables

**Table 1:** Liposomal ADU-S100 formulations with varying lipid compositions and N/P ratios.

Formulation	DOTAP:HSPC:Cholesterol:DSPE-PEG_2000_ (mol%)	Apparent N/P Ratio ^1^	DOTAP:Helper Lipid
F1	45:0:50:5	20:1	1:1
F2	34:11:50:5	15:1	1:2
F3	23:23:50:5	10:1	1:3.5
F4	11:34:50:5	5:1	1:8
F5	6:39:50:5	2.5:1	1:16
F6	34:11:50:10	15:1	1:2
F7	34:11:50:0	15:1	1:2
F8	34:11:50:5	15:1 (MIX Lipo ADU-S100)	1:2
F9	34:11:50:5	Blank liposome	1:2

^1^ Apparent N/P ratio: the molar ratio between the cationic amine (NH3^+^) group in the DOTAP and the anionic phosphate (PO4^3−^) groups in the ADU-S100.

**Table 2:** The EC_50_ values of the IRF3 and NF-κB pathways in the THP-1 Dual cell line treated with free or liposomal ADU-S100.

Formulation	Composition	EC_50_ (µg/mL)	
		IRF3 Pathway	NF-κB Pathway
	Free ADU-S100	3.031	4.839
F1	45 mol% DOTAP, 5 mol% PEG	0.019	0.043
F2	34 mol% DOTAP, 5 mol% PEG	0.026	0.059
F3	23 mol% DOTAP, 5 mol% PEG	0.071	0.122
F4	11 mol% DOTAP, 5 mol% PEG	2.184	3.990
F6	34 mol% DOTAP, 10 mol% PEG	0.090	0.162
F7	34 mol% DOTAP, 0 mol% PEG	0.081	0.104
F8 (MIX lipo ADU-S100)	34 mol% DOTAP, 5 mol% PEG	0.101	0.207

## Data Availability

Not applicable.

## Supplementary Materials

The following supporting information can be downloaded at: https://www.mdpi.com/article/10.3390/pharmaceutics15020638/s1, Table S1: Size distribution and zeta potential of liposomal ADU-S100 formulations with varying amounts of DOTAP and PEGylation; Figure S1: HPLC chromatogram of ADU-S100 and cAMP (internal standard); Figure S2: Flow cytometry gating strategy for analyzing BMDCs and the expression of co-stimulatory molecules; Figure S3: Cell viability assays of liposomal ADU-S100 with varying levels of DOTAP; Figure S4: Production of TNFα (A) and IFNβ (B) in BMDCs after stimulation with free ADU-S100 (5 µg/mL) or liposomal ADU-S100 (0.5 µg/mL) for 24 h; Figure S5: Representative flow plots of CD40, CD80, and CD86 expression after stimulation of BMDCs with PBS and free ADU-S100 (0.5 µg/mL) for 6 h.
