# Supplementary material for: Liposomal Delivery of MIW815 (ADU-S100) for Potentiated STING Activation
Source: Pharmaceutics. 2023 Feb 14;15(2):638. doi: 10.3390/pharmaceutics15020638 (PMC9966736; doi:10.3390/pharmaceutics15020638)
Supplement: Supplementary file 1 [file pharmaceutics-15-00638-s001.zip › pharmaceutics-2078534-supplementary.pdf]

Supplementary Materials

Liposomal Delivery of MIW815 (ADU-S100) for Potentiated STING Activation

Nan Ji [1,†], Minjia Wang [1,†], and Chalet Tan [2,*]

1 Department of Pharmaceutics and Drug Delivery, University of Mississippi, University, MS 38677, USA
2 Department of Pharmaceutical Sciences, University of Tennessee Health Science Center, Memphis, TN 38163, USA.
† These authors contributed equally to this work.
* Correspondence: ctan5@uthsc.edu (C.T.)

Table S1. Size distribution and zeta potential of liposomal ADU-S100 formulations with varying amounts of DOTAP and PEGylation. Results are presented as the mean ± SD (n ≥ 3).

Formulation	DOTAP: HSPC: Cholesterol: PEG_{2000}-DSPE (mol%)	N/P ratio	Size (nm), PDI	Zeta potential (mV)
F1	45:0:50:5	20:1	102.62±3.87 (0.18±0.01)	13.73±1.19
F2	34:11:50:5	15:1	105.30±3.10 (0.17±0.02)	12.53±1.62
F3	22:23:50:5	10:1	100.70±6.77 (0.15±0.05)	12.00±2.56
F4	11:34:50:5	5:1	91.39±9.62 (0.16±0.02)	9.16±2.90
F5	6:39:50:5	2.5:1	87.44±7.72 (0.16±0.03)	5.24±3.11
F6	34:11:50:0	15:1	85.34±6.48 (0.25± 0.03)	46.80±3.97
F7	34:11:50:10	15:1	92.43±6.43 (0.13±0.02)	9.07±3.00
F8 (MIX lipo)	34:11:50:5	15:1	98.30±3.70 (0.14±0.02)	7.73±1.82
F9 (blank lipo)	34:11:50:5	15:1	96.50±4.10 (0.15±0.02)	11.83±1.42

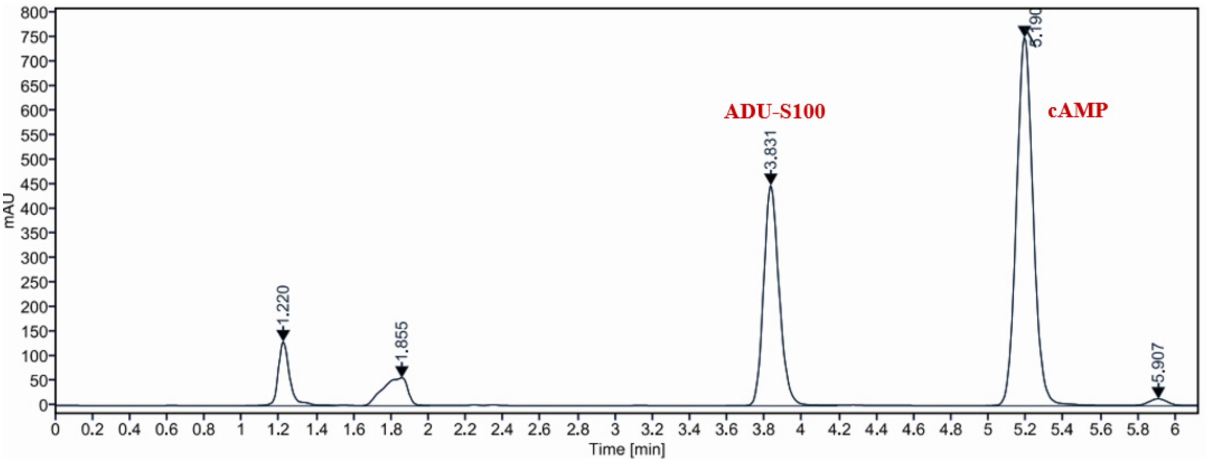

Figure S1. HPLC chromatogram of ADU-S100 and cAMP (internal standard).

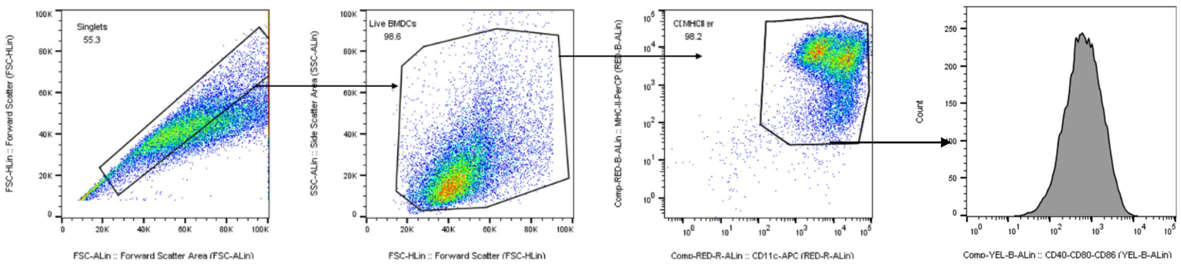

Figure S2. Flow cytometry gating strategy for analyzing BMDCs and the expression of co-stimulatory molecules.

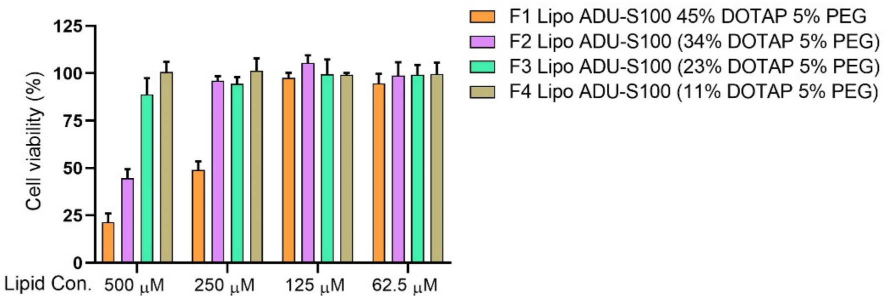

Figure S3. Cell viability assays of liposomal ADU-S100 with varying levels of DOTAP. Human lung carcinoma A549 cells were treated with liposomal AUD-S100 formulations at four lipids concentrations (500, 250, 125, 62.5 μM), which corresponded to four ADU-S100 concentrations (4, 2, 1, 0.5 μg/ml). After a 48-h incubation, the cell viability assay was measured by CyQUANT XTT cell viability kit (Invitrogen) according to the manufacturer's protocol.

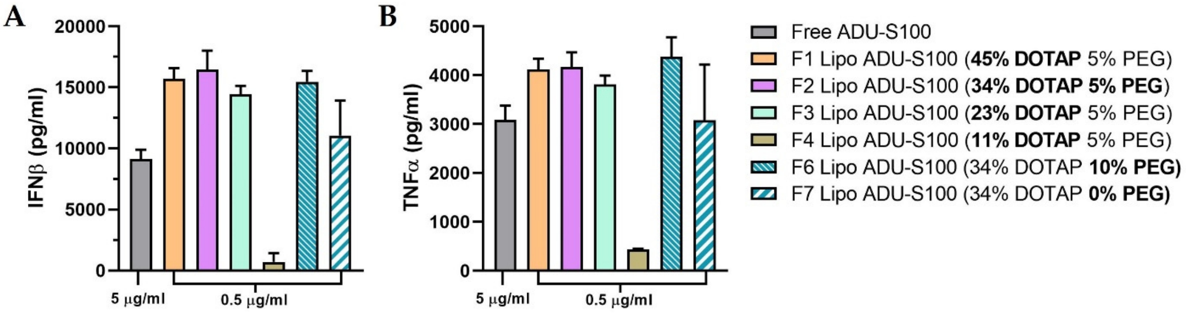

Figure S4. Production of TNFα (**A**) and IFNβ (**B**) in BMDCs after stimulation with free ADU-S100 (5 μg/ml) or liposomal ADU-S100 (0.5 μg/ml) for 24 h. Data are shown as the mean ± SD (n ≥ 3) and are representative of three independent experiments.

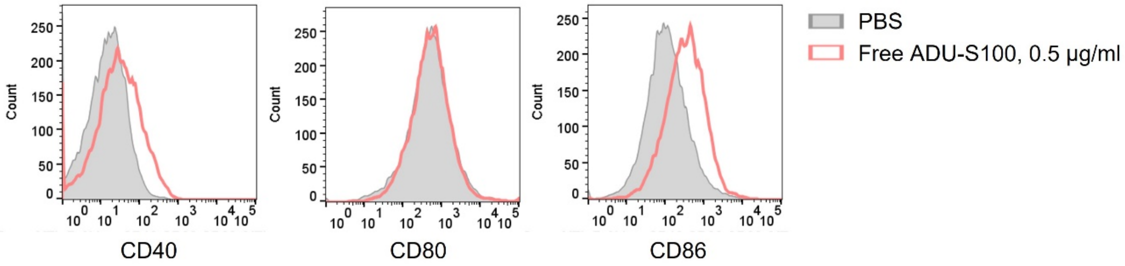

Figure S5. Representative flow plots of CD40, CD80, and CD86 expression after stimulation of BMDCs with PBS and free ADU-S100 (0.5 μg/ml) for 6 h. Data are representative of three independent experiments.
